# Evaluation of Habitat Preferences of Invasive Macrophyte *Egeria densa* in Different Channel Slopes Using Hydrogen Peroxide as an Indicator

**DOI:** 10.3389/fpls.2020.00422

**Published:** 2020-04-30

**Authors:** Takashi Asaeda, M. D. H. Jayasanka Senavirathna, Lekkala Vamsi Krishna

**Affiliations:** ^1^Hydro Technology Institute, Tokyo, Japan; ^2^Institute for Studies of the Global Environment, Sophia University, Tokyo, Japan; ^3^Research and Development Center, Nippon Koei, Tsukuba, Japan; ^4^Department of Environmental Science, Saitama University, Saitama, Japan

**Keywords:** invasive species, environmental stress, oxidative stress, river vegetation, empirical equations

## Abstract

*Egeria densa* is an often-found invasive species in Japan, which has spread widely in the past two decades in rivers where no macrophytes had previously been found. As a result, these ecosystems have now become dominated by *E*. *densa*. The habitat preference for *E*. *densa* colony formation was investigated using the tissue concentrations of hydrogen peroxide (H_2_O_2_: a reactive oxygen species) under varying conditions in rivers and laboratory conditions. The empirical equations that can describe the macrophyte tissue H_2_O_2_ formation under various velocity and light conditions were produced. The H_2_O_2_ concentrations of dark-adapted plants are proportional to the flow velocity, and the surplus H_2_O_2_ concentration in the light-exposed condition corresponded to the photosystems produced H_2_O_2_. When the H_2_O_2_ concentration exceeds 16 μmol/gFW, plant tissue starts to deteriorate, and biomass declines, indicating the critical values required for long-term survival of the plant. The empirically obtained relationships between flow velocity or light intensity and the analysis of H_2_O_2_ concentration for different slopes and depths of channels found that the H_2_O_2_ value exceeds the critical H_2_O_2_ concentration in channels with above 1/100 at around 0.6 m depth. This agrees with the observed results where colonies were not found in channels with slopes exceeding 1/100, and biomass concentration was the largest at depths of 0.6 to 0.8 m. H_2_O_2_ concentration is quite applicable to understanding the macrophyte condition in various kinds of macrophyte management.

## Introduction

The mid-streams of large Japanese rivers were characterized as gravel beds during the post-World War II era. Fine sediment beds were extremely limited. Thus, the ecosystems of gravel beds, characterized by rich hyporheic flows and biota, such as insect larvae and salmonid fish, were maintained for long periods (Hauer et al., 2016). Except for some emergent species, *Phragmites japonica* (Asaeda et al., 2009), almost no submerged macrophyte colonies existed in the main streams of major rivers (Kadono, 2004). Since then, dams and weirs have been frequently constructed and most of the waterways have been regulated. Therefore, almost all the gravel particles introduced upstream are trapped before entering the midstream, thus the supply of gravel to the midstream and downstream is completely curtailed. In addition, gravel was mined for use as construction materials from the 1960s to 1970s. The amount of gravel, therefore, substantially reduced in the midstream, compared to that of previous years (Asaeda and Sanjaya, 2017). In contrast, fine sediment inflows continued from the mid to downstream catchments. They were transported and settled, filling interstices on downstream gravel beds. Thus, the midstream beds are now partially covered with fine sediments that bury stones.

In the past two decades, invasive macrophyte *Egeria densa* began to form colonies in many rivers (Ministry of Lands Infrastructure Transportation and Tourism in Japan [MLIT], 2016). It often covers extensive areas of the channel bed and completely changes the ecosystem there (Collier et al., 1999; Yarrow et al., 2009). Financially, this causes substantial losses to inland fisheries, particularly in the yield of Ayu fish (*Plecoglossus altivelis altivelis*), a grazer of benthic algae (Asaeda et al., 2018). *E*. *densa* was cultivated in aquariums in the early 19th century, but it was disposed into natural freshwater bodies and became naturalized in the 1940s. However, it had not spread into rivers as they were gravelly in those days and were not in a suitable condition to support submerged macrophytes, however, it has been found in some lakes of western Japan since the 1970s (Kadono, 2004). Besides Japan, *E*. *densa*, spread widely in other continents (Champion and Tanner, 2000; Santos et al., 2011). It affected stream ecosystems extremely, retarding flow velocity, increasing sedimentation (Collier et al., 1999), and exile of native species (Santos et al., 2011; Gillard et al., 2017). Therefore, though the effects were particularly eminent in Japanese gravel rivers, the invasion of *E*. *densa* is a worldwide problem.

As several changes occur simultaneously in natural rivers, it is not easy to elucidate the primary reason that prevented earlier macrophyte colonization or their increase today. The habitat preferences of macrophytes are normally evaluated by monitoring their growth rate or biomass (Barko et al., 1991; Riis et al., 2012; O’Hare et al., 2018). However, there are various potentially influential factors in the natural environment and each factor changes from time to time during the period of the macrophytes’ growth. The existing conditions are, thus, considered to be a result of the integrated environmental conditions experienced previously, and the casual observation that is mainly practiced in vegetation management is not necessarily appropriate for evaluating their habitat preference.

In natural water, macrophytes are subjected to environmental stresses, such as flow velocity, high solar radiation, excessive high or low temperature, etc. In cell organelles, then, reactive oxygen species (ROS) are generated based on the intensity of the stresses, photosynthesis and metabolic activities (Zaman and Asaeda, 2013; Asaeda and Rashid, 2017; Parveen et al., 2017a). A part of these ROS is scavenged relatively quickly by antioxidant activities, and the homogeneity of ROS in tissues is maintained by a balance between the ROS and the antioxidants. However, under excessive stress, this balance collapses as oxidative stress surpasses the antioxidant capacity of the plant. The existence of ROS in plant tissues leads to oxidative stress, and when critical levels are exceeded, the plants tend to deteriorate (Sharma et al., 2012; Choudhury et al., 2017). The most common ROS is hydrogen peroxide (H_2_O_2_), which is generated by the superoxide dismutase by in the superoxide (Asada, 2006; Sharma et al., 2012). The H_2_O_2_ concentration is relatively stable and can be easily analyzed chemically (Satterfield and Bonnell, 1995; Zhou et al., 2006). The amount of tissue H_2_O_2_ concentration, therefore, has potential for use as an indicator to monitor the instantaneous environmental stress intensity on macrophytes (Asaeda et al., 2018).

During the daytime, the total amount of H_2_O_2_ generated in plant tissues is, therefore, primarily the sum of the H_2_O_2_ generated in response to environmental stress, photosynthesis, and other nonstress metabolic products. This process can be generalized as the following simple equation:

(1) H2⁢O2(Total)=H2⁢O2⁢(Photosynthesis)+H2⁢O2⁢(Metabolic⁢byproduct⁢and⁢respiration)+∑H2⁢O2⁢(Environmental⁢stress)

Although there are some interactions between the different environmental stressors and opposing trends in some combinations of stressors (Rivero et al., 2014), the share of H_2_O_2_ concentration of stresses is separated from metabolic, respiration, and photosynthesis produced H_2_O_2_ (Mittler, 2002). However, the plant oxidative stress is determined by cumulative H_2_O_2_ content present in the cells regardless of the source.

Several types of stressors are acting on submerged macrophytes in natural rivers. In the relatively steep non-polluted rivers, the major stressors include the mechanical stress introduced by high current velocity/turbulence, solar radiation, and temperature (Yarrow et al., 2009; Riis et al., 2012). As these are based on different physical quantities, it is difficult to compare the magnitude of each stressor on the submerged macrophytes. However, it is possible to differentiate photosynthesis produced H_2_O_2_ from the total accumulated H_2_O_2_ by dark adapting the plants (Asaeda et al., 2018). In addition, when other stresses are eliminated under controlled conditions in the laboratory, it is possible to quantify each type of stress by the produced H_2_O_2._

Considering the facts that, it can be hypothesized that (1) there is a relationship between induced H_2_O_2_ concentration in *E*. *densa* and the intensity of each stress given by the habitat metrics, such as water velocity, temperature, and the solar radiation of the habitat. (2) *E*. *densa* growth reduced and is deteriorated in the condition in which H_2_O_2_ concentration exceeds a threshold value. (3) The habitat metric condition for *E*. *densa*, therefore, remains to be a H_2_O_2_ concentration less than the threshold value. Then, the habitat preference and adaptability of *E*. *densa* is studied in terms of H_2_O_2_ formation under varying riverine conditions and the controlled conditions in the laboratory, focusing on obtaining empirical relationships of factors on the tissue H_2_O_2_ contents.

As the tissue presence of H_2_O_2_ can be used to evaluate the plant condition, and the plant H_2_O_2_ content can be evaluated in a short period, it has the potential to be adopted in macrophyte monitoring practices. In the present study, we focused the H_2_O_2_ production of *E*. *densa* over various field and laboratory conditions. However, the methodology is widely applicable for various types of macrophyte managements, such as the identification of the optimum condition in the endangered species’ restoration, or in the extermination of alien species.

## Materials and Methods

### Field Observations

Several rivers that are highly colonized by *E*. *densa* were selected from the species distribution records in Japan (Ministry of Lands Infrastructure Transportation and Tourism in Japan [MLIT], 2016). In 2016 and 2017, observations were conducted in rivers for location data of *E*. *densa* colonization. Sampling activities were conducted on fine days (days with clear sky and no rain expected) in different seasons from Eno (Go), Saba and Hii Rivers, including their tributaries (Table 1). In each river, the surveys reached approximately 20 to 50 km from the upstream to the downstream area, and five to ten sites where more than one third of the bed was covered with pure *E*. *densa* colonies were selected for the study, including the most upstream colony in the main channel.

**TABLE 1:** River channel data where large colonies of *E*. *densa* were found.

	River or tributary	Distance from the river mouth or conjunction (km)	Channel bed slope	Maximum channel depth at normal water level (cm)	Approximate depth of *E*. *densa* colony (cm)
1	Yahagi River	45.2	1/800	120	30–100
2	Yoshii River	83.5	1/200	50	40–50
3	Asahi River, Nakatsui River Tributary	14.6	1/270	50	40–50
4	Ashida River	79.1	1/210	40	40
5	Ashida River, Takaya River Tributary	0.5	1/1800	40	30–40
6	AshidaRver, Mitsugi River Tributary	9.3	1/220	50	40–50
7	Eno (Gono) River, Mainstream^a^	89.3	1/400	130	50–100
8	Eno (Gono) River, Tajibi River Tributary^a^	0.5	1/120	80	40–80
9	Eno (Gono) River, downstream of Haji Dam^a^	92.5	1/250	60	30–60
10	Eno River, upstream of Haji Dam	160.8	1/280	50	30–50
11	Eno (Gono) River, Saijo River tributary	5.6	1/320	40	30–40
12	Eno (Gono) River, Joge River Tributary	40.5	1/150	40	30–40
13	Ohta River, Misasa River Tributary	3.1	1/400	70	50–70
14	Hii River, Small Tributary	5.0	1/400	50	30–50
15	Takatsu River	57.3	1/160	50	50
16	Takatsu River, Tsuwano River Tributary	18.3	1/180	40	40
17	Saba River, Shimaji River Tributary^b^	10.8	1/190	110	50–110

*^*a*^Sampling was conducted on May 24 and 25, 2016; September 16, 2016; April 18, 2017; and June 11–13, 2017. ^*b*^Sampling was conducted on May 25 and 26, 2016; June 17, 2016; September 17, 2016; April 18, 2017; June 13–15, 2017; and August 7–9, 2018. ^*c*^Sampling was conducted on October 11, 2016.*

At each sampling site, there were several *E*. *densa* patches, and each patch was composed of several plants. Thus, more than five samples were collected from overlying shoots of different plants of a same patch in light-exposed (under natural conditions) and dark-adapted conditions to differentiate photosynthesis generated H_2_O_2_ from environmental factors and metabolism induced H_2_O_2_ of tissues. The dark exposure treatment was performed by placing a black plastic sheet (3 m × 3 m) floating over the *E*. *densa* colonies for 30 min. The 30 min pre-dark period was determined from laboratory experiments, which were conducted to determine the optimum pre-darkness duration. The plastic sheets were tied to fixed metal poles that were inserted in the riverbed, allowing the sheets to float on the water surface without causing mechanical disturbances to the macrophytes or altering the water flow. The PAR intensity under the sheet was found to be zero. The light-exposed samples were collected adjacent to the darkness treated samples. The collected samples were put in resealable plastic bags and quickly stored in a cool box containing dry ice until they were transferred to the laboratory to be stored at −80°C. Biomass was obtained from a 50 cm × 50 cm quadrant of each sampling point.

At each sampling point, the water velocity was measured with an ultrasonic velocimeter and recorded for more than 1 min (Tokyo Keisoku Co. Ltd., Japan), at 20% (reference velocity) and 80% (depth of the colony) of the total water depth. Turbulence velocity component was calculated as a root mean square deviation from the mean velocity, from the velocity record. Photosynthetically active radiation intensity (PAR intensity) in the water was measured with a portable quantum flux meter (Apogee, MQ-200, United States) at 10 cm depth intervals.

On October 23, 2018, the lateral configuration of a channel was investigated to derive the effect of the *E*. *densa* colony on the environment, including the distributions of depth, sand depositions thickness, *E*. *densa* biomass, particle size, and the *E*. *densa* burying condition in trapped sediments at a point 45 km upstream from the river mouth of the Yahagi River. During the summers of 2016, 2017, and 2018, surveys were conducted from the upstream to the downstream areas in other rivers where the existence of *E*. *densa* was recorded, then the locations and the depth of the rivers were recorded and the channel slope was obtained from a topographic map [Geographical Survey Institute of Japan, the (Gsi), 2018].

### Laboratory Experiments

The pre-darkness period in the field observation was investigated as follows. The apical cuttings with an average length of 10 cm were obtained from the stock culture and planted in two tanks (50 cm × 35 cm × 35 cm) with thoroughly washed commercial river sand (90% of <0.2 mm particle size; washed using tap water several times until all organic materials are washed away and finally, washed using distilled water to further remove nutrients). In each tank, twenty *E*. *densa* cuttings were planted and maintained in a temperature-controlled room maintained at a constant 23 ± 3°C temperature. Each tank was exposed to approximately 100 μmol/m^2^/s PAR with a 12 h/12 h light and dark period. Nutrients were supplied via a 5% Hoagland nutrient solution. After a two-month acclimatization period, one of the two tanks was covered entirely by a black plastic sheet, providing darkness. *E*. *densa* tissues from different samples were collected at 10 min intervals for 2 h and then collected at 6, 12, and 24 h. Light-exposed samples were collected, simultaneously. To avoid the stress of cutting, the tissues were collected from fresh tips during each sampling activity. The experiment was conducted in triplicates with different samples. The H_2_O_2_ concentrations of tissues were then analyzed. The H_2_O_2_ concentration of *E*. *densa* gradually declined with the dark duration, taking the lowest value at 30 min, then slightly increasing later for all cases. Therefore, in the field experiment, 30 min of darkness was adopted for the dark-adapted samples.

The effect of temperature on H_2_O_2_ generation was investigated, using four tanks similar to those in the previous experiments. After an acclimatization period of 2 months, the temperature regimes of the tanks were set to 10, 15, 25, and 30°C, respectively, using an aquarium water temperature controlling system (Aquarium cooler ZC-100α, Zensui Corporation, Japan). Light intensities, 220, 320 and 680 μmol/m^2^/s were obtained with the combination of several LED lamps. Then, a 5 m long flume equipped with a straightening plate at the upstream end was used to check the velocity effect. The central part was lightened with 200 μmol/m^2^/s of PAR intensity by a LED lamp. The velocity was adjusted at 23 cm/s to maintain the low turbulence intensity condition, less than 2 cm/s of turbulence velocity, following Asaeda et al. (2018), and the normal flow velocity of the *E*. *densa* habitat (Champion and Tanner, 2000). The temperature conditions were maintained for 7 days, and plants were sampled for the chemical analyses. The experiment was conducted in triplicates with different samples for each condition.

### Chemical and Biomass Analyses

The tissue H_2_O_2_ was estimated colorimetrically using spectrophotometry (Asaeda et al., 2018). Plant chemicals were extracted into ice-cold phosphate buffers (50 μmol/L, pH 6.0) by crushing approximately 100 mg of the plant in the presence of polyvinylpyrrolidone (PVP). The extractions were centrifuged at 5000 *g* for 15 min at 4°C. The enzyme extraction of 750 μL was then mixed with 2.5 mL of 0.1% titanium sulfate in 20% (v/v) H_2_SO_4_, and the mixture was centrifuged at 2500 *g* for 15 min at 20°C. The optical absorption at a wavelength of 410 nm was measured using spectrophotometry (UV-1200, UV-Visible Spectrophotometer, Shimadzu, Japan), and the H_2_O_2_ concentrations (μmol/gFW) were estimated using a standard curve.

The dry weight of biomass was estimated by oven drying the collected biomass samples at 70°C for 72 h or until the weight became stable. The dried biomass was weighed and expressed in units of gDW/m^2^.

### Statistical Comparison

The linear or power law (for solar radiation intensity) correlation between parameters was tested by Pearson’s correlation analysis and the statistical significance between observations were tested with Student’s *t*-test. The statistical comparisons of field data were performed to obtain the relationship between the H_2_O_2_ content and the external factors (such as velocity, turbulence velocity, light intensity, biomass, and depth), and the relationship between the factors (turbulence and mean flow). For H_2_O_2_ concentration and velocity or temperature relationship, statistical comparisons were performed for the different study sites and/or sampling time groups, which have different temperatures and solar radiations, to obtain the interaction between stresses and the H_2_O_2_ concentration. The gradient of the regression line was obtained for the whole set of data in the relation between each stress component and H_2_O_2_ concentration. Then, for each study site and sampling time groups, statistical analysis was conducted to check the significance of the regression. For the relationship between H_2_O_2_ concentration and light intensity, the power law regression of the excessive H_2_O_2_ concentration of the light exposed samples and dark-adapted samples was conducted to obtain the light intensity at zero H_2_O_2_ concentration. Then the data scattering was compared with the standard deviation.

All the statistical tests were performed using IBM SPSS Statistics Version 25. The regression lines and equations were obtained using the inbuilt regression function feature of Microsoft Excel 2016.

## Results

**FIGURE 1:**
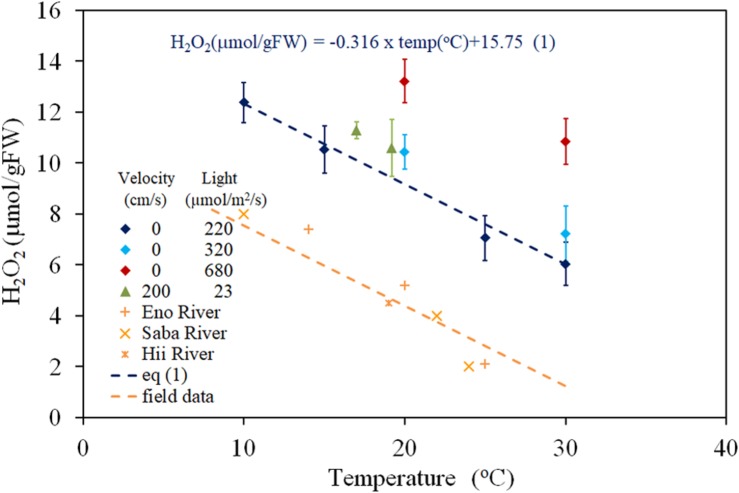
The concentration of H_2_O_2_ in *E*. *densa* tissue as a function of water temperature. The error bars represent the standard deviation (*n* = 3). Eno, Hii, and Saba Rivers data are values extrapolated to zero velocity of the regression line with –0.316 (μmol/gFW cm/s) gradient, shown by dashed lines in Figure 2.

The relationship between H_2_O_2_ concentration and water temperature, obtained from both laboratory experiments (PARs were 220, 320, and 680 μmol/m^2^/s with 0 cm/s of velocity; 200 μmol/m^2^/s of PAR and 23 cm/s of flow velocity), and dark-adapted condition of the field observation (PAR = 0 μmol/m^2^/s) is shown in the Figure 1. The recorded temperatures of field studies ranged between 10 and 25°C depending on the sampling seasons and rivers, and the fluctuations were observed to be 1–2°C in the same sampling condition groups. Thus, the extrapolated regressed lines of each group to zero velocity were used here (Figure 1). H_2_O_2_ concentration has a negative correlation with temperature in the 10 to 30^*o*^C temperature range and was regressed to lines with a gradient of −0.316 μmol/gFW/degree, for all light intensity groups. The regression equations are shown in Figure 1 compared to observed data (*R* = 0.982, *P* < 0.01 for 220 μmol/m^2^/s PAR; *R* = 0.963, *P* < 0.01 for field observation, 0 μmol/m^2^/s PAR). There was no overlapping among data from different groups. Thus, the effect of interaction between temperature and light intensity is sufficiently small.

**FIGURE 2:**
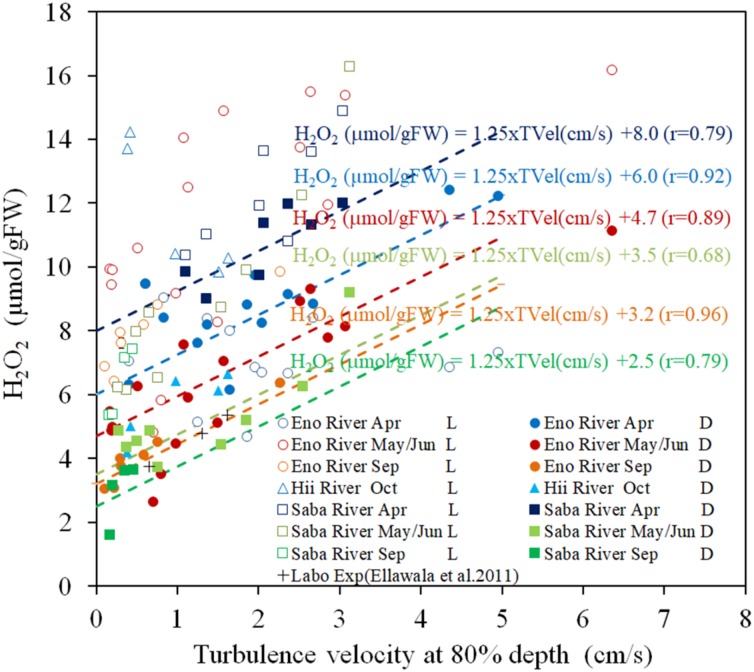
The concentration of H_2_O_2_ in *E*. *densa* tissue as a function of turbulence velocity at 80% depth in light-exposed (L) and dark-adapted (D) conditions. The “Labo Exp” represents data from the laboratory experiment (Ellawala et al., 2011). The dashed lines indicate the linear regressed lines with the gradient of 1.25 μmol/[gFW (cm/s)] of the dark-adapted conditions of the same colored symbols of as those of the sampling rivers and seasons, respectively. H_2_O_2_ is given in units of μmol/gFW, and while the *TVel* (turbulence velocity) are in cm/s. The dates May, Sep (September), and Oct (October) represent data obtained in 2016 while Jun (June) and Apr (April) represent data of 2017. Eno, Saba, and Hii represent Eno, Saba, and Hii rivers including their tributaries, respectively.

Figure 2 presents the H_2_O_2_ contents of the field observation samples with respect to turbulence velocity. Under light exposure, the H_2_O_2_ contents are always higher than those in the corresponding dark-adapted samples by 5–10 μmol/gFW. However, the scattering was greater than that in the dark-adapted samples. Dark-adapted samples are composed of different temperature groups, which depend on the sampling time and rivers.

The H_2_O_2_ contents of dark-adapted samples were highly correlated with the turbulence velocity, with a gradient of 1.25 μmol/[gFW (cm/s)] (*R* = 0.796, *P* < 0.01). The regression lines with the same gradient are shown in Figure 2 for the dark-adapted samples of different sampling time and river groups, compared to observed data. For each group, the H_2_O_2_ contents were highly regressed to the lines (*R* = 0.917, 0.885, 0.964 for April, May/June and September sampling at the Eno River, respectively, 0.76 for the Hii River, and 0.84, 0.68, and 0.63, respectively, for the sampling during each season, respectively, at the Saba River for all *P* < 0.01). This finding indicates that the H_2_O_2_ concentration dependence on turbulence velocity is independent of temperature.

**FIGURE 3:**
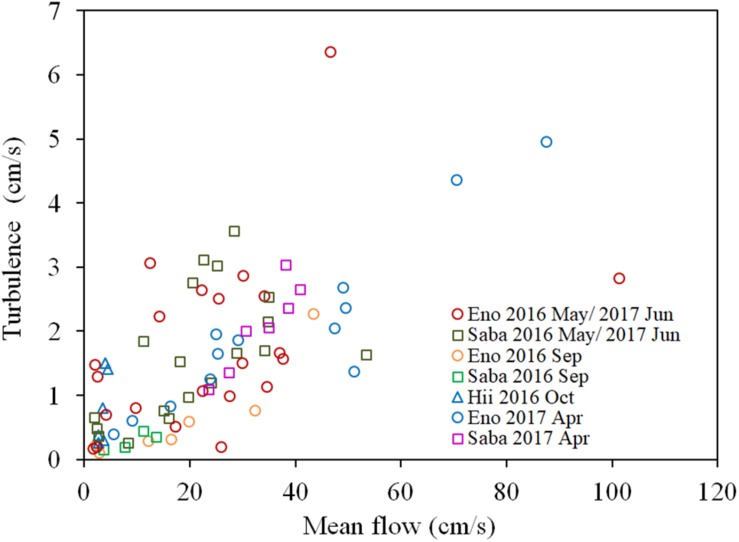
Relationship between the turbulence velocity and the mean flow velocity of each sampling site of different rivers. The sample collected month is represented by the month indicated in the legend. Eno, Saba, and Hii represent Eno, Saba, and Hii rivers, including their tributaries, respectively.

**FIGURE 4:**
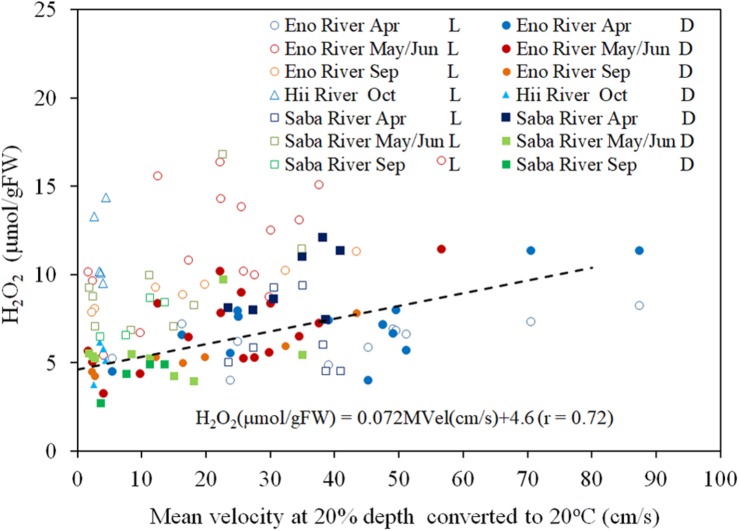
The concentration of H_2_O_2_, converted to 20°C, in *E*. *densa* tissue as a function of the mean velocity at 20% deep thin light-exposed (L) and dark-adapted (D) conditions. The dashed line indicates the linear trend of the dark-adapted condition. The dates May, Sep (September), and Oct (October) represent data obtained in 2016 while Jun (June), Apr (April) represent data of 2017. Eno, Saba, and Hii represent Eno, Saba, and Hii rivers including their tributaries, respectively. H_2_O_2_ is given in units of μmol/gFW, and the *MVel* (mean velocity) is in cm/s.

There is a significant positive correlation between the mean flow and turbulence velocity (*R* = 0.722, *P* < 0.01), as shown in Figure 3. Also, the correlation between the H_2_O_2_ content of dark-adapted samples with the mean velocity is also positive and significant (Figure 4, *R* = 0.571, *P* < 0.01). The relationships can be explained with linear regression equations [Eq. (2) for the 0–60 cm/s mean flow velocity range]. The line in Figure 4 included the cases in which the turbulence intensity was particularly high because of the large gravel beds. Although these values provide slightly higher H_2_O_2_ concentrations compared to those at normal sites, the relatively proportional relationship with turbulence velocity indicates that the mean flow velocity is available as a reference of the mechanical stress due to flow velocity (Asaeda et al., 2017).

(2) $$H_{2}\,O_{2}\,\left(\mu \,m\,o\,l/\text{gFW}\right)=0.072\,M\,V\,e\,l\,\left(\text{cm}/\text{s}\right)+4.6$$

**FIGURE 5:**
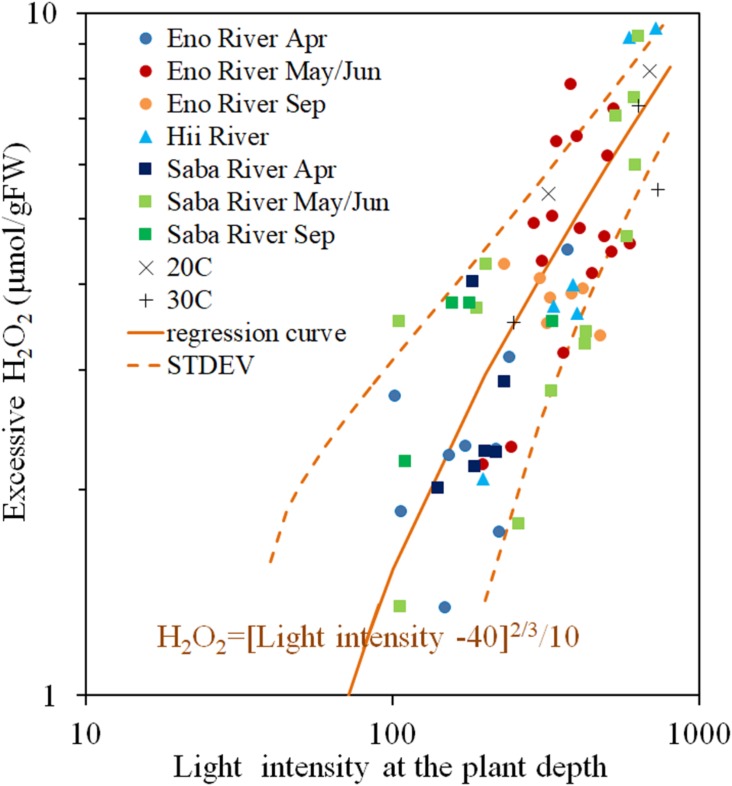
Photosynthesis-produced H_2_O_2_ concentration (Excessive H_2_O_2_) of *E*. *densa* tissue as a function of light intensity. The sample collected month is represented by the year and the month indicated in the legend. Eno, Saba, and Hii represent Eno, Saba, and Hii Rivers, including their tributaries, respectively. When the light intensity is lower than 40 μmol/m^2^/s, the excessive H_2_O_2_ concentration was 0 μmol/gFW. The regression curve in the excessive H_2_O_2_ concentration and the excessive light intensity from 40 μmol/m^2^/s condition is postulated by a rigid line, and the standard deviation is shown by the dashed line. “Light” is the light intensity at the sample depth (μmol/m^2^/s), and the excessive H_2_O_2_ concentration, *H*_2_*O*_2_*rad* (*Temp*), is expressed in μmol/gFW. The “*H*_2_*O*_2_*rad*” denotes the light induced H_2_O_2_ content. The Exp 20^*o*^C and Exp 30^*o*^C represent the excessive H_2_O_2_ concentration quantified in experiments under 20 and 30°C controlled temperatures.

The H_2_O_2_ concentration of the light-exposed samples fluctuated heavily, but always exceeded the value of the corresponding dark-adapted samples. The excessive H_2_O_2_ content is light induced H_2_O_2_ content, postulated as a function of light intensity in Figure 5. There is a positive correlation between the light intensity and the H_2_O_2_ concentration, however, the increasing rate of the excessive H_2_O_2_ decreases with increasing light intensity. When the light intensity is lower than 40 μmol/m^2^/s, the excessive H_2_O_2_ was nearly 0, similar to experimental results obtained by Hussner et al. (2010) and Rodrigues and Thomaz (2010). Therefore, power low regression analyses were conducted for the excessive H_2_O_2_ concentration with respect to the surplus light intensity from 40 μmol/m^2^/s, as Eq. (3) in the figure (*R* = 0.738, *P* < 0.05). Although, the scattering is large, most of the data are distributed within the standard deviation from the Eq. (3) (1.57 μmol/gFW) without any systematic deviation regardless of rivers and sampling seasons, where the solar radiation intensity ranged from 40 to 600 μmol/m^2^/s, and temperature from 10 to 25^*o*^C.

(3) $$H_{2}\,O_{2}\,\left(\mu \,m\,o\,l/\text{gFW}\right)={\left[L\,i\,g\,h\,t\,I\,n\,t\,e\,n\,s\,i\,y-40\right]}^{\frac{2}{3}}/10$$

### Diurnal H_2_O_2_ Variation of *E. densa*

**FIGURE 6:**
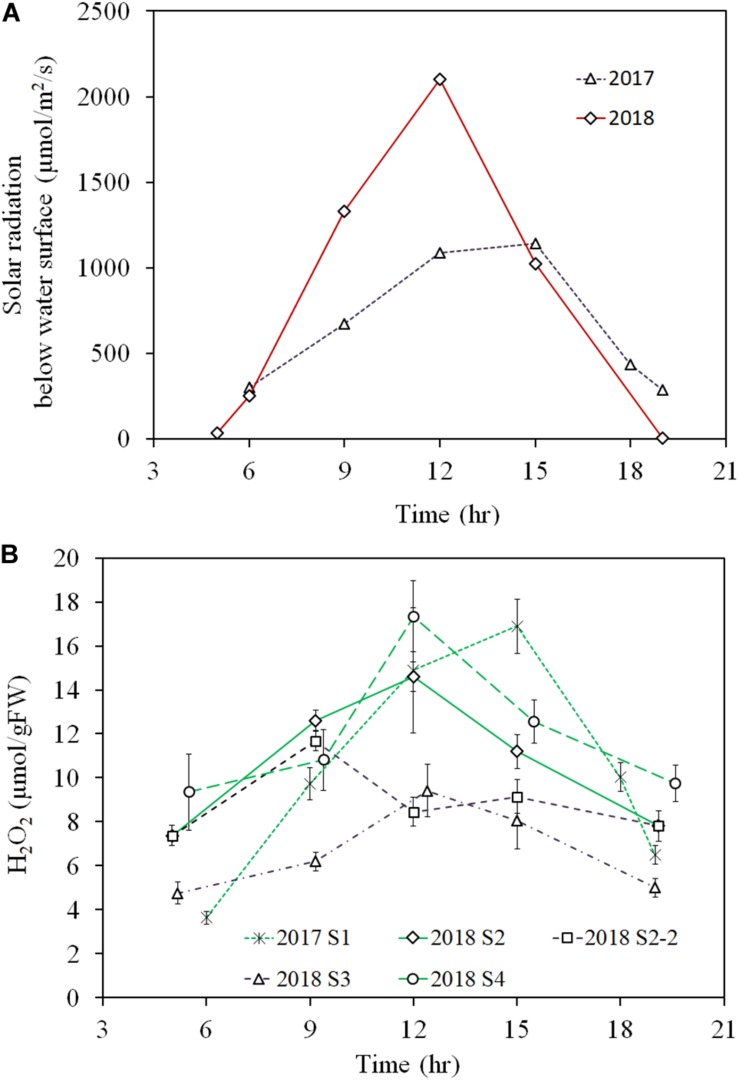
Diurnal variation of light intensity just below the water surface of 2017 and 2018 observations **(A)** and *E*. *densa* tissue H_2_O_2_ concentration **(B)**, observed in the years 2017 (moderate radiation), and 2018 (excessive radiation). 2017 S1 represent the healthy shoots of 2017 observation. 2018 S2-2 and 2018 S3 represent degraded shoots under high solar radiation exposure at shallow water (<10 cm deep) in 2018, and 2018 S2 and 2018 S4 represent healthy shoots exposed to moderate radiation in deep depth sites (S2 30 cm deep and S4 50 cm deep) of the observation. The error bars represent the standard deviation.

In the diurnal observation, the light intensity during the 2018 sampling was higher for the 2018 observation day (∼1500 μmol/m^2^/s at the shoot height) compared to that for the 2017 observation day (<1000 μmol/m^2^/s at the shoot height). This high solar radiation intensity condition continued for several weeks before the 2018 observation day, while rainy or cloudy conditions had persisted for several weeks before the 2017 observation day. Therefore, samples from the 2018 observation were exposed to high solar radiation for several weeks, as opposed to samples from the 2017 observation (Figure 6A).

The diurnal variation of tissue H_2_O_2_ concentration followed the diurnal solar radiation intensity in 2017 and for healthy samples at 2018 S2 and S4 in 2018 (Figure 6B). Then, during the day, the H_2_O_2_ concentration rose up to nearly 16 μmol/gFW by noon and then, declined in the afternoon with the decline in solar radiation. In contrast, the values were substantially lower for the degraded samples in the 2018 observation. Then, samples at 2018 S2-2 and 2018 S3, showed less than 10 μmol/gFW of H_2_O_2_ concentration around noon. *E*. *densa* colonies remained healthy in the 2017 observation, while in the 2018 observation, shoots close to the water surface were degraded and appeared to be starting to die at S2-2 and S3.

**FIGURE 7:**
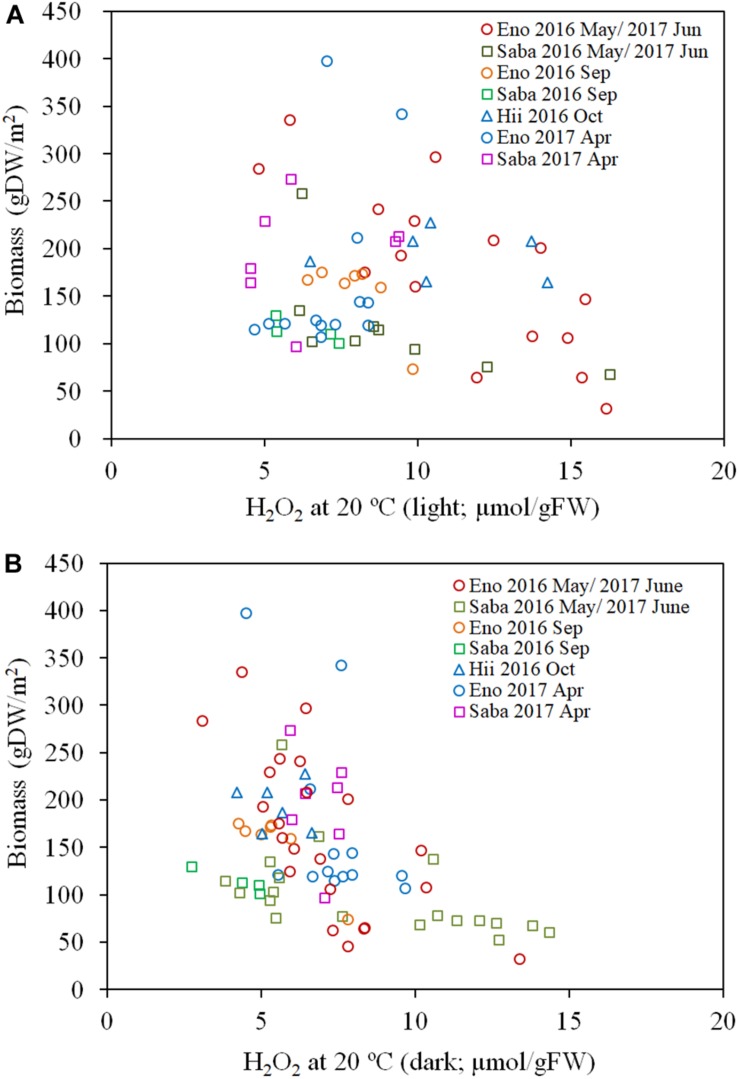
Biomass of *E*. *densa* as a function of light exposed H_2_O_2_ concentration **(A)** and dark-adapted H_2_O_2_ concentration **(B)**. The H_2_O_2_ concentration was corrected to 20°C. The sample collected month is represented by the year and the month indicated in the legend. Eno, Saba, and Hii represent Eno, Saba, and Hii rivers, including their tributaries, respectively.

**FIGURE 8:**
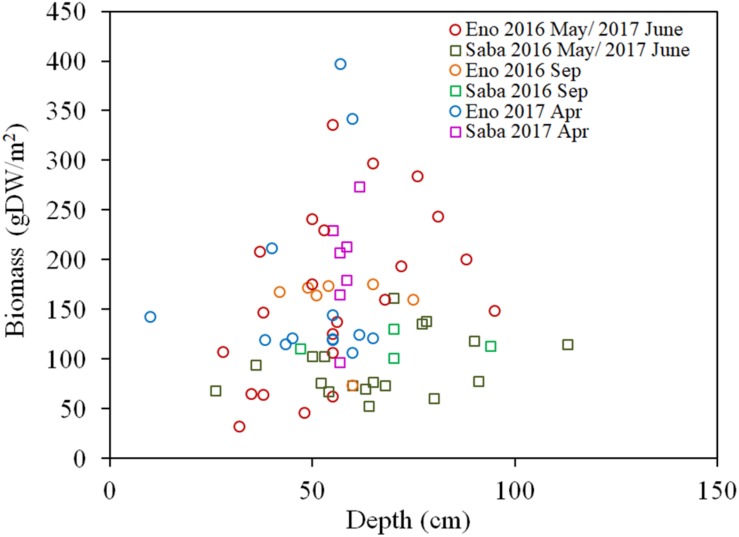
Biomass of *E*. *densa* as a function of water depth. The sample collected month is represented by the month indicated in the legend. Eno and Saba represent Eno and Saba rivers, including tributaries, respectively.

As per Figures 7A,B, the H_2_O_2_ content for both the light-exposed and dark-adapted samples were negatively correlated with the biomass (*R* = −0.474, *P* < 0.01 for light-exposed and *R* = −0.504, *P* < 0.01 for dark-adapted). It was observed that there were no samples with an H_2_O_2_ content exceeding the 16 μmol/gFW range. The approximate channel slope of the river in which large colonies of *E*. *densa* were formed in the running water was between 1/120 and 1/1800. No *E*. *densa* colony was found in the further upstream reaches, with channels steeper than 1/100, unless weirs were constructed to regulate the water flow (Table 1). The stress on plants generated by the flow velocity is more intensified at deeper sites; however, a high biomass concentration was found in deeper zones in the channel rather than in the shallow zones. The biomass of the colonies was highly correlated with the depth of the water column, peaking at a depth of 80 cm, and gradually declining as the depth increased further (Figure 8). The biomass distribution exhibited a common trend irrespective of the river or the observation site.

## Discussion

### Environmental Stressors on *E. densa*

There are several types of stressors acting on submerged macrophytes in natural rivers. As these are based on different physical quantities, it is difficult to compare the magnitude of the effect of each stressor on the submerged macrophytes. The difference in H_2_O_2_ concentrations between the continuous light-exposed and 30 min pre-shaded samples differentiate the stress-induced H_2_O_2_ from photosynthesis generated H_2_O_2_. Based on the outcome of the laboratory experiment, we developed a relationship between temperature and the H_2_O_2_ generation in plant tissues. When the fractions of H_2_O_2_ corresponding to photosynthesis and temperature effects were eliminated from the continuous light-exposed samples of field studies, the observed trend of H_2_O_2_ was similar to the result of the oscillating grid laboratory experiment, in which the amount of H_2_O_2_ in macrophyte tissues was proportional to the root mean square velocity of the turbulence as is shown in Figure 2 (Asaeda et al., 2017).

Under the zero-turbulence velocity, the tissue H_2_O_2_ concentration corresponds to a combination of photosynthesis, metabolic activities, and environmental stresses. The difference between light and dark treatment experiments distinguishes the amount of H_2_O_2_ generated by photosynthesis from the remaining stressors. Further, the variation in solar radiation intensity during the day is reflected in the H_2_O_2_ concentrations, which varied according to the light intensity from approximately 2 to 10 μmol/gFW (Figure 5). There were parallel relationships between the H_2_O_2_ concentration and the temperature for different light intensity groups with the same velocity (Figure 1), which explains the independency of H_2_O_2_-temperature relationship trend from the light intensity but simultaneously elevates the trend due to excessive H_2_O_2_ production (Figure 5). The temperature-H_2_O_2_ dependency linear relationship provides the temperature generated H_2_O_2_ concentration, which is reportedly around ∼10 μmol/gFW in the *E*. *densa* habitat temperature range of 10–35^*o*^C, in the present observation and previous reports (Hanamoto and Ikushima, 1988.; Yarrow et al., 2009; Gillard et al., 2017).

H_2_O_2_ concentration of dark-adapted samples had linearly increasing trends with respect to turbulence velocity, regardless of different sampling months and river groups, which differentiate the temperatures (Figure 2). The H_2_O_2_ concentration extrapolated to zero velocity had the same relationship with the temperature dependency on the H_2_O_2_ concentration (Figure 1). Thus, the increasing rate of the H_2_O_2_ concentration with respect to turbulence effects indicates the turbulence induced H_2_O_2_.

These H_2_O_2_-solar radiation and H_2_O_2_-temperature relationships exhibit almost the same trends for photosynthetic rates obtained by the outdoor experiments with different temperatures and light intensities (Riis et al., 2012). Therefore, the H_2_O_2_ relationship with the light intensity and temperature conditions, can be considered as common trends for *E*. *densa*. In the practical application, H_2_O_2_ concentration can be applicable to determine the different types of stressors in the same manner and to compare their relative magnitudes.

Compared to the H_2_O_2_ induced by metabolic activities, which is ∼4 μmol/gFW given in the field experiments of dark-adapted samples at zero velocity, in the relatively steep non-polluted rivers, the major stressors include the mechanical stress introduced by high current velocity/turbulence, solar radiation, and temperature. The river water quality is relatively good, and there is no salinity in the midstream of Japanese rivers. However, in eutrophic water, organic matter accumulates at the bottom of stagnant zones, which creates an anoxic zone in the sediment layer; however, the bottom sediment anoxia contributed only ∼5 μmol/gFW of H_2_O_2_ (Parveen et al., 2017a). As for the biotic stress, toxic strains of cyanobacteria, *Microcystis*, for instance, generate only 1.5 μmol/gFW of H_2_O_2_ (Amorin et al., 2017). Therefore, the amount of H_2_O_2_ generated by photosynthesis, temperature and flow velocity is relatively large compared to other stresses, and these stresses are considered as major stresses, which control its colonization.

The contribution of each stress to the total H_2_O_2_ of the plant can be distinguished with this method and can be adapted to determine the total level of environmental stress on macrophytes. The combination of different stresses sometimes imposes two opposing demands on the plant (Choudhury et al., 2017) or interact each other. However, the parallel relationship among the major stresses in natural rivers, namely, solar radiation, flow velocity, and water temperature and the interactive effects seems to be sufficiently small. The reason for the relatively lower interactive effects among stressors is not clear.

H_2_O_2_ is generated by the surplus number of electrons. In the photosynthesis process, the surplus amount of electrons are generated on the thylakoid membrane by strong energy (Asada, 2006), while the consumption of electrons is decreased under low temperature due to the suppressed CO_2_ fixation by the inactivation of Rubisco in Calvin cycle (Nishiyama and Murata, 2014), or the mechanical damage of organelles in turbulent flow (Atapaththu et al., 2015). Therefore, the sites that can cause the electron surplus is different between stresses.

### Threshold Condition of *E. densa* Mortality

*Egeria densa* colonies of 2017 observation remained healthy, while in the 2018 observation, shoots close to the water surface were degraded and appeared to be starting to die. The intensive oxidative stress caused by high solar radiation for several days should be the reason for the degradation of 2018 colonies. The results indicate the depression of plant metabolism owing to solar radiation exceeding the tolerable levels. When the H_2_O_2_ concentration became higher than 16 μmol/gFW in hypoxia and hydrogen sulfate exposure experiments for *E*. *densa*, plants deteriorated, and the total chlorophyll concentration and the H_2_O_2_ concentration substantially declined compared to other samples (Parveen et al., 2017a,b,c). Also, with the exposure of Fe, *E*. *densa* exhibited lowest growth rate, chlorophyll content and photosystem efficiency at around 16 μmol/gFW of H_2_O_2_ content and beyond the level, healthy plants did not exist (unpublished data). Same as the other observations, the 2018 observations show the H_2_O_2_ level of colonies peaked beyond the 16 μmol/gFW during the daytime. Therefore, the H_2_O_2_ concentration of 16 μmol/gFW can be considered as a critical value for the survival of *E*. *densa*, regardless of the types of stressors. Exceeding the threshold level, would lead to the deterioration of plants due to oxidative damage.

### Empirical Expression of Habitat Preference and Colonizable Conditions Simulation

The H_2_O_2_ concentration of macrophytes can be used to explain the expected macrophyte distribution in a river. When Eq. (4) is considered, the H_2_O_2_ generated by each stressor can be expressed as follows:

The total H_2_O_2_ concentration (*H*_2_*O*_2_*tot*) at a particular temperature (*Temp*) is given by:

(4) H⁢O2⁢t2⁢o⁢t⁢(T⁢e⁢m⁢p)=H⁢O2⁢r2⁢a⁢d⁢(T⁢e⁢m⁢p)+H⁢O2⁢v2⁢e⁢l⁢(T⁢e⁢m⁢p)+H⁢O2⁢m2⁢e⁢t⁢(T⁢e⁢m⁢p)

where *H*_2_*O*_2_*rad* is the H_2_O_2_ generated by solar radiation exposure, *H*_2_*O*_2_*vel* is the H_2_O_2_ generated by the flow velocity, and *H*_2_*O*_2_*met* is the H_2_O_2_ generated by metabolism.

The light intensity at a particular depth of water, *z*, can be calculated using Eq. (5) (Middelboe and Markager, 1997):

(5) I=I⁢e0⁢x⁢p⁢(-k⁢z)

where *k* is the attenuation coefficient in water, which was 0.035 (/cm) in the observed rivers. The solar-radiation-induced H_2_O_2_ content has an increasing relationship with the light intensity, given by Eq. (3). Considering Eq. (3), the H_2_O_2_ concentration generated by the solar radiation under a particular temperature (within the temperature range 10–30°C), therefore, can be expressed as Eq. (6):

HO2r2ad(Temp)=[Ie0xp(-kz)-40]/2/310forIe0xp(-kz)≥40μmol/m/2s;

(6) HO2r2ad(Temp)=0forIe0xp(-kz)<40μmol/m/2s.

Direct mechanical stress is generated by turbulence rather than the mean flow (Atapaththu et al., 2015; Asaeda and Rashid, 2017; Asaeda et al., 2017); however, there is a close relationship between these two quantities, particularly in straight uniform channels with uniform roughness. It is assumed that the mean velocity *U* (m/s) under the uniform flow is empirically given by Manning’s law [Eq. (7)]:

(7) $$U=\frac{1}{n}\,R^{2/3}\,S^{1/2}$$

where *R* is the hydraulic radius, approximately given by the depth “*H*” (m), the channel bed slope “*S*,” and Manning’s roughness coefficient “*n*.” The effects of longitudinal configuration, vegetation, etc., are added in “*n*.” Therefore, considering Eq. (2), the H_2_O_2_ accumulation due to velocity stress and metabolism at a particular temperature (within the temperature range 10–30°C) can be expressed as follows (Eq. 8):

(8) $$H_{2}\,O_{2}\,v\,e\,l\,\left(T\,e\,m\,p\right)=0.072\,\frac{1}{n}\,H^{2/3}\,S^{1/2}+4.6$$

Subsequently, the total H_2_O_2_ concentration within the temperature range 10–30°C can be given as follows (Eq. 9):

$$H_{2}\,O_{2}\,\left(T\,e\,m\,p\right)={\left[I_{0}\,e\,x\,p\,\left(-k\,z\right)-40\right]}^{2/3}/10+0.072\,\frac{1}{n}\,H^{2/3}\,S^{1/2}+4.6$$

for *I*_0_ exp (-*kz*) ≥ 10 μmol/m^2^/s;

(9) $$H_{2}\,O_{2}\,\left(T\,e\,m\,p\right)=0.072\,\frac{1}{n}\,H^{2/3}\,S^{1/2}+4.6$$

for *I*_0_ exp (-*kz*) < 10 μmol/m^2^/s.

**FIGURE 9:**
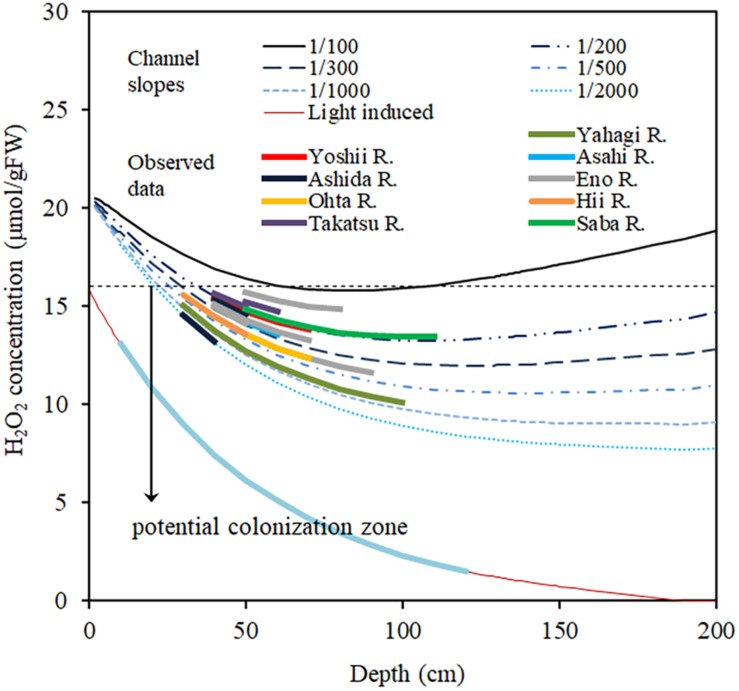
Simulated H_2_O_2_ concentration in the plant body of *E*. *densa* changes with the water column depth for different channel slopes and actual slope data of several Japanese rivers. The line “Light-induce” represents the H_2_O_2_ concentration induced by light and change with the depth. The downward arrow “potential colonization zone” represents the depth zone of different channel slopes suitable for *E*. *densa* colonization.

Figure 9 shows the simulated H_2_O_2_ concentration generated by using Eq. (9), for different channel slopes and depths. *I*_0_ is assumed to be 2000 μmol/m^2^/s, as the highest solar radiation experienced in the observation area on a fine day during summer and when the canopy top was assumed to be located at the 80% depth, which is the average canopy height of the observed *E*. *densa* colonies. Manning’s roughness coefficient for the channels is approximated at 0.08. Channel slope was obtained from the topographic map (ArcGIS, 2019, Esri, New York Street, Redlands, California). The target area was characterized by steep basins, and the difference in elevation between the channel bed and the riparian zone is nearly constant thus the riverbed gradient is nearly the same as that of the riparian zone. The simulated results of Figure 9 are consistent with the observed data of the rivers. The H_2_O_2_ concentration generally declines with depth, due to declining light intensity. In the steep channel, the H_2_O_2_ concentration increases again with a further increase in depth as the flow velocity rises. On gentler slopes, due to the lower flow velocity, H_2_O_2_ concentration is lower, and there is a wider range of depths in which *E*. *densa* colonies potentially form. However, the shallow depths become unsuitable for colonization of *E*. *densa* due to the higher light intensity.

### *E*. *densa* Colonization in Rivers

In rivers steeper than 1/100, the upstream to midstream areas are originally filled with gravel or boulders, and fine sediment beds are rare as they are easily flushed away during flooding. The flow velocity depends on the channel slope, depth, and roughness. Roughness is determined by the bed sediment size as well as other factors related to the channel configuration, such as longitudinal morphology and bars (Parker and Peterson, 1980). The formation of *E*. *densa* colonies poses a physical disadvantage due to the high flow velocity and high light intensity as well as the high turbulence caused by the gravel bed as long as gravel sediments are supplied. However, when the gravel supply is decreased, the gravel-to-sand transition is extended further upstream (Singer, 2008), and the sand bed area increases in the former gravel zones. Sand is transported mostly as a suspended sediment load along the channel. *E*. *densa* communities accumulate suspended sediments efficiently, as they have a complex, dense stem structure that is widely distributed over the bed with thicker stems and denser whorls compared to similar native species, *Hydrilla verticillata*, and common native submerged species, *Elodea* sp., *Myriophyllum spicatum*, and *Potamogeton crisps* (Sand-Jensen, 1998; Vermaat, et al. 2000; Statzner et al., 2006). At the same time, water intake weirs have been frequently constructed along rivers in the last 30 years (personal communication). Then, stagnant water was produced in the upstream zone. Thick *E*. *densa* colonies were developed in the upstream area of many weirs in the observation (data are not shown). With the *E*. *densa* dispersal ability via fragmentation, it spread increasingly into the downstream (Casati et al., 2000, 2002; Redekop et al., 2016). Then, the dense shoot morphology leads to the formation of many sandy patches in the former complete gravel beds of the downstream. When fine sediments are supplied, it is easier to fix roots and take nutrients from the ground than from the stony bed (Barko et al., 1991). Buried by a sediment layer, the *E*. *densa* complex shoots reinforce the sand layer, which is otherwise easily washed away by floods. Once the *E*. *densa* colonies develop, the sandy spaces increase widely on the gravel bed, accommodating more macrophytes, including other species. Subsequently, the ecosystem changes to a macrophyte-dominated ecosystem. A similar phenomenon was observed, after the occurrence of a large flood in the Yahagi river (in July 2017), sandy sediments accumulated in colonies burying *E*. *densa* up to 20 to 30 cm on a bed that was originally gravelly (Supplementary Figure 1). The *E*. *densa* biomass was relatively dense, up to 150–400 gDW/m^2^, in these sites, as the accumulated sand layer was reinforced with *E*. *densa* stem structures. This layer was thus rigid and could not be easily flushed away during moderate floods (Supplementary Figure 2). In contrast to the original gravel bed, the sandy surface was smooth, and turbulence generation was also reduced. Further, in field observations, an accumulation of fine sediments was always found inside the *E*. *densa* colonies of all rivers.

## Conclusion

The high concentrations of H_2_O_2_ introduced by high flow velocity and high solar radiation in summer inhibited the formation of large colonies in the gravel channel, owing to the high oxidative stress. The accumulation of H_2_O_2_ in *E*. *densa* showed a significant relationship for both flow velocity and solar radiation. The critical H_2_O_2_ concentration to maintain a healthy population of *E*. *densa* can be considered as 16 μmol/gFW, which corresponds to the termination of biomass accumulation. Under the strongest solar radiation on summer days, the H_2_O_2_ level often exceeds the critical condition, leading to the deterioration of *E*. *densa* and ultimately, the H_2_O_2_ concentrations decline as the plant tissues start to deteriorate. H_2_O_2_ concentrations of *E*. *densa* were estimated for channels with different slopes and different depths. The H_2_O_2_ concentration is higher than the critical value in shallow water and increases in steeper channels, exceeding the critical value at a channel slope larger than 1/100. Once colonized, *E*. *densa* accumulates sandy suspended sediment efficiently and creates a preferable environment for further colonization. The present methodology can be applied to predict the area that can be conveniently colonized by *E*. *densa* within a short time period, which has been determined based on a prolonged monitoring activity.

## Data Availability Statement

All datasets generated for this study are included in the article/Supplementary Material.

## Author Contributions

TA conceived the study. TA and LV performed the experiments. TA and MS analyzed and interpreted the data, and wrote the manuscript. All authors reviewed the final version of the manuscript.

## Conflict of Interest

The authors declare that the research was conducted in the absence of any commercial or financial relationships that could be construed as a potential conflict of interest.
