# Supplementary material for: Evaluation of Habitat Preferences of Invasive Macrophyte Egeria densa in Different Channel Slopes Using Hydrogen Peroxide as an Indicator
Source: Front Plant Sci. 2020 Apr 30;11:422. doi: 10.3389/fpls.2020.00422 (PMC7204913; doi:10.3389/fpls.2020.00422)
Supplement: Supplementary file 1 [file Data_Sheet_1.docx]

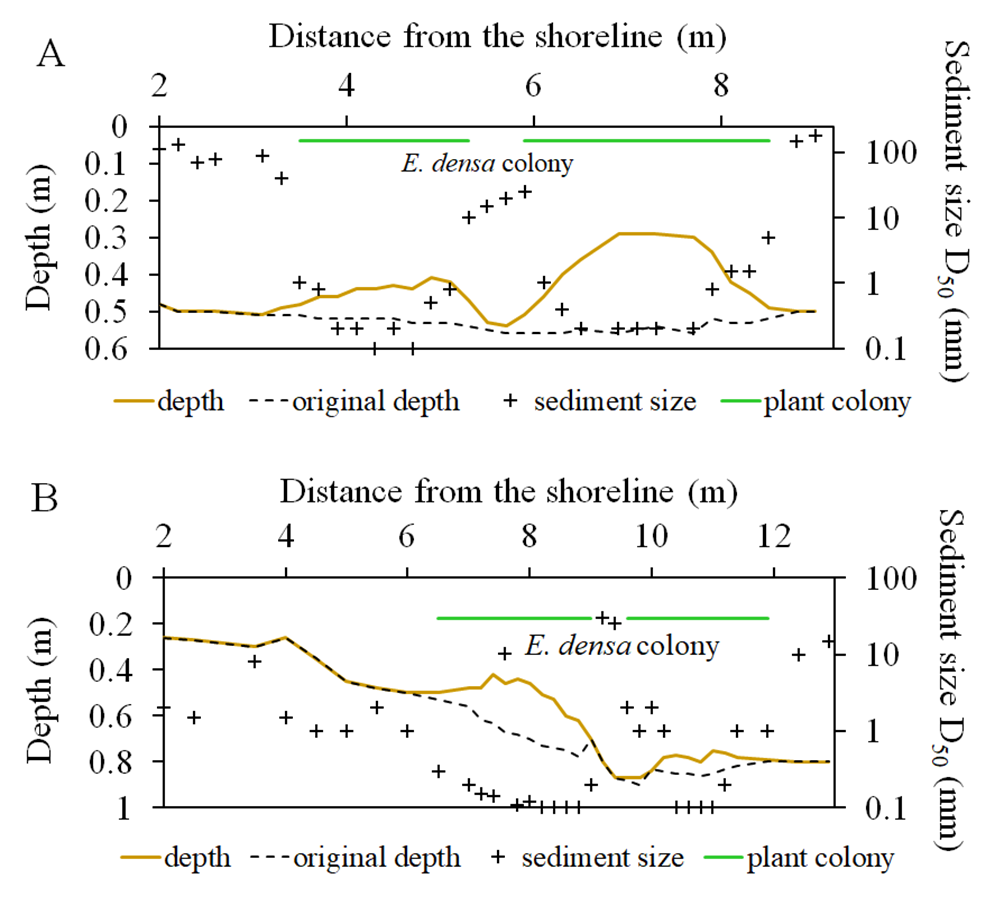


Supplementary figure 1. Sediment accumulation after a flood in the Yahagi river observed in two cross sections (A, B). The depth and original depth represent the depth after the flood and before the flood, respectively. The sediment size represents 50% of particle sizes. The plant colony represents the area where E. densa was found on the cross-section.
