# Supplementary material for: Evaluation of Habitat Preferences of Invasive Macrophyte Egeria densa in Different Channel Slopes Using Hydrogen Peroxide as an Indicator
Source: Front Plant Sci. 2020 Apr 30;11:422. doi: 10.3389/fpls.2020.00422 (PMC7204913; doi:10.3389/fpls.2020.00422)
Supplement: Supplementary file 2 [file Data_Sheet_2.docx]

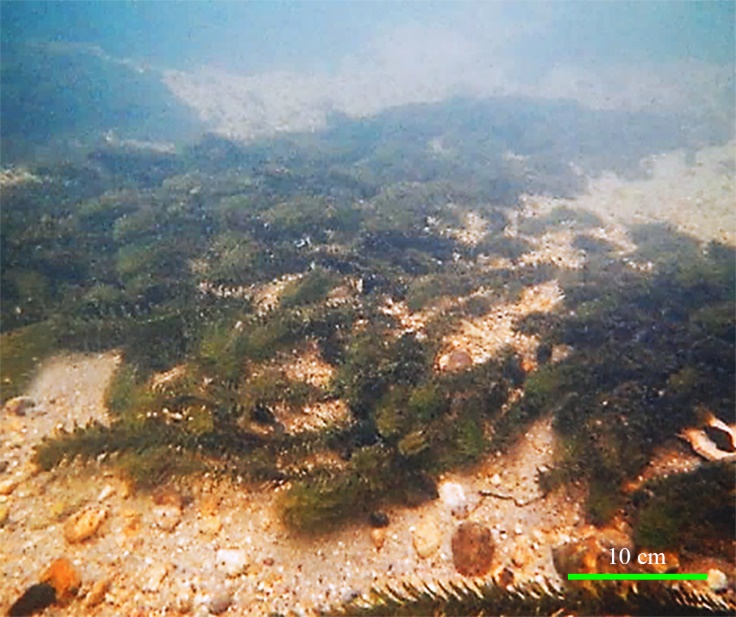


Supplementary figure 2. A representative image of the Yahagi riverbed occupied by *E. densa* at a depth of 80–100 cm.
